# Feasibility and preliminary effect of anabolic steroids in addition to strength training and nutritional supplement in rehabilitation of patients with hip fracture: a randomized controlled pilot trial (HIP-SAP1 trial)

**DOI:** 10.1186/s12877-021-02273-z

**Published:** 2021-05-20

**Authors:** Signe Hulsbk, Thomas Bandholm, Ilija Ban, Nicolai Bang Foss, Jens-Erik Beck Jensen, Henrik Kehlet, Morten Tange Kristensen

**Affiliations:** 1 Physical Medicine and Rehabilitation Research Copenhagen (PMR-C), Department of Physiotherapy and Occupational Therapy, Copenhagen University Hospital, Hvidovre, Denmark; 2 Department of Orthopedic Surgery, Copenhagen University Hospital, Hvidovre, Denmark; 3 Department of Clinical Research, Copenhagen University Hospital, Hvidovre, Denmark; 4 Department of Clinical Medicine, University of Copenhagen, Copenhagen, Denmark; 5 Department of Anesthesiology, Copenhagen University Hospital, Hvidovre, Denmark; 6 Department of Endocrinology, Copenhagen University Hospital, Hvidovre, Denmark; 7 Section for Surgical Pathophysiology 7621, Copenhagen University Hospital, Copenhagen , Denmark

**Keywords:** Rehabilitation, Strength training, Nutritional supplement, Anabolic steroid, Hip fracture, Physical therapy, Physical function, Body composition

## Abstract

**Background:**

Anabolic steroid has been suggested as a supplement during hip fracture rehabilitation and a Cochrane Review recommended further trials. The aim was to determine feasibility and preliminary effect of a 12-week intervention consisting of anabolic steroid in addition to physiotherapy and nutritional supplement on knee-extension strength and function after hip fracture surgery.

**Methods:**

Patients were randomized (1:1) during acute care to: 1. Anabolic steroid (Nandrolone Decanoate) or 2. Placebo (Saline). Both groups received identical physiotherapy (with strength training) and a nutritional supplement. Primary outcome was change in maximal isometric knee-extension strength from the week after surgery to 14weeks. Secondary outcomes were physical performance, patient reported outcomes and body composition.

**Results:**

Seven hundred seventeen patients were screened, and 23 randomised (mean age 73.4years, 78% women). Target sample size was 48. Main limitations for inclusion were not home-dwelling (18%) and cognitive dysfunction (16%). Among eligible patients, the main reason for declining participation was Overwhelmed and stressed by situation (37%). Adherence to interventions was: Anabolic steroid 87%, exercise 91% and nutrition 61%. Addition of anabolic steroid showed a non-significant between-group difference in knee-extension strength in the fractured leg of 0.11 (95%CI -0.25;0.48) Nm/kg in favor of the anabolic group. Correspondingly, a non-significant between-group difference of 0.16 (95%CI -0.05;0.36) Nm/Kg was seen for the non-fractured leg. No significant between-group differences were identified for the secondary outcomes. Eighteen adverse reactions were identified (anabolic=10, control=8).

**Conclusions:**

Early inclusion after hip fracture surgery to this trial seemed non-feasible, primarily due to slow recruitment. Although inconclusive, positive tendencies were seen for the addition of anabolic steroid.

**Trial registration:**

Clinicaltrials.gov NCT03545347.

**Supplementary Information:**

The online version contains supplementary material available at 10.1186/s12877-021-02273-z.

## Introduction

Patients with a hip fracture are a vulnerable group with high morbidity and mortality. Sustaining a hip fracture leads to large strength deficits [1, 2], causing loss of function, disability and further falls [3,5]. As such, a hip fracture often result in loss of independence, change of residence, more fractures and high mortality rates [6,9], and constitutes a substantial economic burden to the health care system [10, 11]. Although positive effects on mobility of structured exercise interventions including strength training are reported [12,15], these interventions alone are insufficient to overcome the major long-term negative impact of a hip fracture on physical function [7]. Thus, it has been argued to investigate the effect of multimodal interventions including muscle-enhancing medicine [4, 9, 16, 17].

A Cochrane Review (2014) evaluated the effect of anabolic steroids in rehabilitation following hip fracture surgery on functional outcome and adverse events [18]. Positive tendencies were identified, but due to high risk of bias, further trials were suggested [18].

Consequently, and based on existing knowledge on rehabilitation following hip fracture [12, 13, 15] and review recommendations [4, 9, 12, 17, 18], we investigated an early multimodal intervention consisting of anabolic steroid, nutritional supplement and exercise, to enhance short and long term outcomes after a hip fracture.

### Purpose

The aim of this pilot trial was to investigate the feasibility and preliminary effect of a 12-week intervention consisting of anabolic steroid in addition to physiotherapy and protein-rich nutritional supplement on knee-extension strength and function at 14-weeks follow-up after hip fracture surgery. Hypotheses are described in the protocol [19].

## Methods

### Trial design

This is the primary trial report for the HIP-SAP1 trial. A randomized (1:1), blinded, single-center, placebo-controlled, two-armed, parallel-group, superiority pilot trial. The trial was approved by the Capital Regions Research Ethics Committee (H-18004495) and the Danish Medicine Agency (EudraCT: 2017001543-13) and registered with the Danish Data Protection Agency, Journal no.: AHH-2017-090, I-Suite No.: 05980. It adhered to the principles of ICH-GCP and was monitored by a local Good Clinical Practice (GCP) Unit. Reporting of the trial follows the CONSORT checklist [20], and the intervention is described according to the TIDieR checklist [21]. Pre-registration at ClinicalTrials.gov, registration number NCT03545347 (04/06/2018). The trial protocol was published December 23, 2019 [19].

### Changes to method after trial commencement

The inclusion period was extended with 1year due to slow recruitment. Nonetheless, the trial was prematurely discontinued due to slow recruitment. All other changes have been reported previously [19].

### Participants

Patients admitted to the Hip Fracture Unit, at the Orthopedic Department, Hvidovre Hospital, University of Copenhagen was screened for eligibility from 5th June 2018 to 24th February 2020. Sampling method was consecutive, though screening was discontinued during trial staffs absence. A full list of eligibility criteria has been published previously [19]. Briefly, patients had to be aged 60 or above, prefracture home-dwelling, with indoor walking ability and without cognitive dysfunction (disoriented, dementia, delirium). Eligible patients were addressed at the ward 14days post-surgery. Full oral and written information was provided by the project coordinator (PhD student and physiotherapist with 12years experience within hip fracture rehabilitation). Patients were offered at least 24h to consider participation and had the opportunity of having a relative or other person accompanying for further information. Patients who agreed to participate signed an informed consent form.

### Intervention

Patients were randomized (1:1) to one of two arms: 1. anabolic steroid (INT) or 2. placebo (CON). Both groups followed identical physiotherapy and nutritional supplement programs. A detailed intervention description has been published [19]. Below is a summery.

#### Trial medication

##### Active arm (INT)

Every 3weeks the patients received intramuscular injections of nandrolone decanoate (Deca-Durabolin 50mg/ml produced by Aspen). First injection was administered at baseline and last injection at week 12. Women received 50mg; men with total testosterone 11nmol/l received 100mg, and men with total testosterone <11nmol/l received a dose of 200mg.

##### Placebo arm (CON)

Following the same intervals as for the active agent, patients received a placebo injection of 1ml Sodium Chloride 9mg/ml (produced by Fresenius Kabi). The injection was administered at the same site as the active agent. The product has no medical therapeutic effect.

#### Nutritional supplement

Two daily nutritional drinks were offered during hospital admission as standard procedure. The protein-rich nutritional supplement was planned to account for at least 35% of the patients daily protein requirement. The recommendations for geriatric patients with acute disease is 1.21.5g/kg bodyweight/day [22]. The standard used at the hip fracture unit is 1.35g/kg bodyweight/day. The protein-rich nutritional supplement is a liquid containing 18g milk-based protein pr. bottle (Nestl Resource 2.0+fiber). Based on the standard used in this trial, patients received 2 bottles per day for 12weeks.

#### Physiotherapy

Physiotherapy was started on postoperative day 1 and included functional exercises such as transfers and walking, as well as exercise therapy primarily aimed at lower extremities. An exercise guide of 12 exercises was handed out and progressed individually [1]. After discharge, patients were referred for physiotherapy in the municipality. The patients received physiotherapy 1h twice a week, up to and including the 12th week after inclusion in the trial. The training session consisted of warm up, functional training, balance training, lower limb exercises and progressive strength training. Two strength training exercises were mandatory (knee-extension and leg press) and performed according to a standardized protocol with 3 sets of each exercise. In the first 2weeks the number of repetitions was 15 with an intensity of 15 repetition maximum (RM), hereafter 2weeks of 12 repetitions with 12 RM and for the remaining 8weeks 10 repetitions with 10 RM [23]. The physiotherapist logged the load, number of repetitions and pain for each set during the session [19] and assisted the patient in progressing the load on a set to set basis if possible, or at least from session to session.

### General trial treatment procedures

Patients included in the trial followed the departments standard procedures for surgery [24], anesthesia and peri-operative care. Standard perioperative care includes D-vitamin and calcium supplement dependent on the patients individual level. After enrollment, baseline testing was carried out by the project coordinator. Due to the extensive test battery, baseline testing often extended over 2days, within post-operative day 310. Patients were randomized after baseline testing. Hereafter, the first injection of the trial solutions was administered by a dedicated nurse. Throughout the trial, weekly telephone calls were conducted by the project coordinator to ensure and monitor compliance as well as detect potential adverse events. The patient visited the hospital every 3rd week, where blood tests was carried out, safety parameters assed and trial medication administered. The project coordinator and dedicated nurse undertook the assessments and medication administration. Further, nutritional supplement covering the following 3weeks were handed out. Patients were offered free transportation.

### Feasibility outcomes

The following feasibility aspects were assessed: Number of eligible patients, inclusion rate per month, feasibility and suitability of outcome measures, the acceptability to the patients of the treatments, adherence to the treatment, retention to the scheduled controls and follow-up, and number and severity of adverse events.

### Outcomes of effectiveness

Blinded outcome assessment was conducted at baseline and at follow-up (week 14) by the project coordinator. Outcomes and time of assessment are described in detail in the published protocol [19]. Below is a short description.

#### Primary outcome

Change in maximal isometric knee-extension strength (Nm/Kg) in the fractured limb from baseline to 14-week follow-up was measured using a belt fixated handheld dynamometer (Commander Muscle Tester; JTech Medical Utah, USA) [1, 23, 25]. The test is conducted as described in the protocol with the patient seated on the bedside, hips, and knee joint angle in 90 flexion and hands placed on the mattress for support [19].

#### Secondary outcomes

Performance measures, patient reported outcomes (PROMs), measures of body composition, hormone levels and lipid profile are described in eMethods in Additionalfile1 and published previously [19].

### Safety parameters

Safety parameters were assessed at baseline, 3,6,9,12,14weeks after inclusion.

The following blood tests were assessed: Hemoglobin, hematocrit, creatinine, carbamide, sodium (Na+), potassium (K+), calcium, INR (P-Coagulation), liver tests, PSA, glucose. Safety thresholds were defined for the following 3 parameters: Hematocrit (safety threshold: Values >0.50); liver tests (safety threshold: If liver test values are >3 times the upper limit of normal); PSA (safety threshold: If PSA increases to more than 50%). Other safety parameters were: Blood pressure, facial hirsutism (Ferriman-Galwey hirsutism score,2 face related items) [26], hoarseness, edema in non-fractured leg, falls. If values exceeded the safety thresholds the treatment with Deca-Durabolin was discontinued. Further, if women experienced displeasing androgenic side effects, treatment was discontinued.

Adverse events (AE) and reactions (AR) including severity and expectedness was recorded throughout the trial period in accordance with European guidelines [27] as described in the protocol [19].

### Sample size

Sample size calculation for the primary outcome; change in knee-extension strength of the fractured limb, were made to detect a between-group difference in the change score of 0.2Nm/kg in favor of the intervention group using Lehrs formula with an SD of 0.22Nm/kg [1, 19]. Based on these estimates, 20 patients were needed in each group using a standard of 80% power and type 1 error rate of 5%. To allow for a 20% dropout rate, 48 patients were planned for inclusion.

### Randomization and allocation

The patients were randomly assigned using a 1:1 allocation ratio. Block randomization (blocks of 2 and 4) was performed and patients were stratified for type of fracture and sex. The allocation sequence was computer generated (random number generator) by a qualified person not involved in the trial. The allocation sequence was retained in a locked cabinet by the person generating the sequence. To ensure allocation sequence concealment, sequentially numbered, opaque, sealed envelopes were used. When a patient entered the trial, the coded envelope was broken by the nurse injecting the trial medication and the envelope was retained by the nurse.

### Blinding procedure

The patients, the healthcare providers, intervention deliverers, data collectors, outcome assessors were all blinded to group allocation. The only person not blinded were the dedicated nurse drawing the envelope and injecting the trial medication.

### Statistical analyses

Descriptive statistics are used to present baseline characteristics. Primary and secondary outcomes are presented as mean (SD) for the sake of simplicity, although normality of distribution is difficult to assess when dealing with small samples. Mean within-group and between-group differences are reported with 95% confidence intervals (CI) and analyzed using a Two sample t-test or Wilcoxon rank sum depended on our best judgement of normality of distribution of the change score. The primary analysis involved all randomly assigned patients with data (available cases, *n*=21) and is here referred to as modified intention-to-treat analysis. Due to the small sample size, missing data was not imputed. The level of significance was set at *p*<0.05.

Secondary per-protocol analyzes were conducted for the primary outcome, excluding patients with less than 75% adherence to training sessions and 80% received injections (in the protocol 100% adherence to injections was stated, which seems unrealistic high, and has been corrected). Since intake of the nutritional supplement was low, no per-protocol analysis was conducted based on the nutritional intake.

## Results

### Recruitment and feasibility

**Fig. 1:**
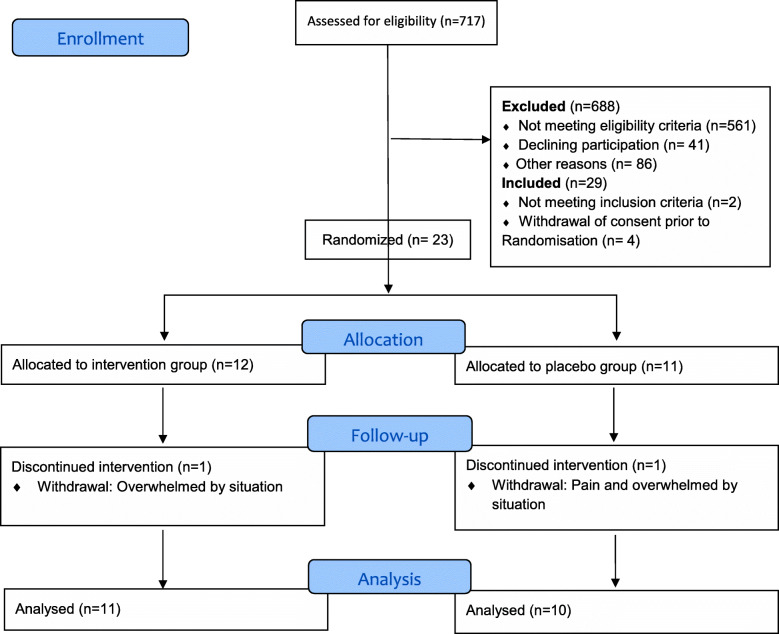
Participant flow chart

Out of 717 screened patients, 29 were included from 6th of June 2018 until 24th February 2020 equivalent to 89weeks (Fig.1). Inclusion was discontinued for 23weeks due to trial staff absence, resulting in an actual inclusion period 16.5months, equivalent to an inclusion rate of approximately 1.8 patients per month. Reasons for non-participation are presented in eTable1 (Additionalfile1), and the two most dominant reasons were not home-dwelling (18%) and cognitive dysfunction (16%). The number of patients declining to participate in the trial was 41, and the most common reason was Overwhelmed and stressed by situation (37%) (eTable2, Additionalfile1). Thus, only 23 patients could be randomized; 12 patients were allocated to INT and 11 to CON (Fig.1). One patient in each group dropped out within the first 2weeks and both declined participation in the follow-up assessment.

### Baseline data

Mean age of the randomized participants was 73.4 (6.7, range 6285) years and 78% were women. In comparison, the mean age of 717 screened patients were 78.3 (12.2) with 66% women. Participants in general had a high prefracture functional level and 91% were discharged home after median 8 (79) days hospitalization. No important differences were identified between INT and CON at baseline (Table1).

**Table 1:** Baseline characteristics of randomized participants (*n*=23)

	Intervention (*n*=12)	Control (*n*=11)
Women n (%)	9 (75)	9 (82)
Men n (%)	3 (25)	2 (18)
Age (years), mean (SD)	73.5 (5.9)	73.4 (7.7)
Body weight (kg), mean (SD)	74.5 (12.4)	77.4 (18.5)
American Society of Anesthesiologist Grade (ASA), mean (SD)	2.1 (0.67)	1.9 (0.30)
Number of pre-existing diagnoses ^a^, mean (SD)	2.9 (2.5)	2.6 (1.1)
Fracture type, n (%)		
Intracapsular	9 (75)	7 (64)
Extracapsular	3 (25)	4 (36)
Type of surgery, n (%)		
2 pins	0	1 (9)
Hemi/total arthroplasty	8 (67)	6 (55)
Dynamic hip screw	1 (8)	1 (9)
Intra medullar hip screw	3 (25)	3 (27)
Living alone, n (%)	7 (58)	6 (55)
Prefracture homecare	1 (8.3)	1 (9.1)
New Mobility Score, 09 points (prefracture), mean (SD)	8.6 (0.8)	8.5 (1.0)
Walking aid indoor, (prefracture), n (%)	0	1 (9)
Walking aid outdoor, (prefracture), n (%)	2 (17)	2 (18)
Discharged home, n (%)	11 (92)	10 (91)
Length of stay (days), median (q1-q3)	8 (69)	8 (79)

^a^Retrieved from hospital chart

### Adherence

#### Medication

Six out of the 21 participants did not receive all 5 injections of the trial solutions; 5 where in the INT and 1 in the CON. Of the 5 in the INT, 1 stopped after 2 injections due to covid-19 and the risk of getting infected by contact to hospital staff, 1 stopped after 2 injections due to a non-related event (myocardial infarction pre-existing coronary stenosis), 1 stopped after 2 injections because of increased liver parameters (classified as related), 1 stopped after 3 injections due to increased perspiration and facial hirsutism (related) and 1 missed 1 injection due to slightly increased PSA value (related). Further, 1 in the CON stopped after 3 injections due to increased liver parameters. Summed up, adherence to injections was 87%.

#### Exercise

The 21 patients exercised in 9 different rehabilitation centers in the uptake area of the hospital. Due to Covid-19 lockdown, 3 patients discontinued the planned exercise intervention in the municipality. These patients were instructed in home exercises (sit to stand, steps/stairs, hip exercises for abductors and extensors) and encouraged to stay as active as possible given the extraordinary situation. However, walking outside their home was restricted to a minimum. Generally, adherence to the municipality-based physiotherapy was excellent with 91%, and with an average of 21.3 (2.3) exercise sessions offered by the municipalities for the remaining 18 participants. Correspondingly, attendance to exercise sessions were 19.4 (2.1), while 1.8 (1.2) sessions were canceled by participants. Adherence to the progressive strength exercises were good, although 5 participants paused their knee-extension exercises due to pain for 1 to 4 sessions. Two of these participants also paused leg press simultaneously. During the first strength training session load-values varied a lot, since the therapist had to find the right load-level, therefore the load progression for the first 2weeks (15RM-period) is calculated from the 2nd to the 4th session. Training loads progressed satisfactorily within the different RM levels, as shown in eTable3, additionalfile1.

#### Nutritional supplement

Average consumption of nutritional supplement was 61% of the planned 168 drinks, and with no significant difference between-groups (INT 58.5% vs CON 63.4%). Two participants did not want to drink the supplement at all. Nine out of 21 participants consumed more than 75%. The most frequent reason for non-consumption was loss of appetite, nausea, dislike taste and reflux.

#### Hospital controls

Only 1 participant did not attend 3 of the scheduled controls due to Covid-19 lock-down (participants own decision), but attended the final follow-up. However, some patients expressed that getting out of the house and back and forth to the hospital was strenuous, during the first hospital visits. On the contrary, many patients expressed gratitude and felt good taken care of, due to the extra controls.

#### Feasibility and suitability of outcome measures

The extensive number of measurements was time-consuming and exhausting to many of the participants at baseline and therefore testing often took place over 2days. Still, high completeness of data suggest that it was feasible. The New Mobility Score (NMS), The Short Falls Efficacy ScaleInternational (FES-I), Geriatric Depression Scale (GDS) and Mini Nutritional AssessmentShort Form (MNA-SF) showed some ceiling effect. Some of the PROMs were a bit overlapping, and in future studies GDS could be left out as depression and anxiety to some extend are included in EQ-5D. DEXA-scanning during the first week after surgery was challenging for participants, as mobility was limited and they were restricted by pain.

### Primary outcome

Knee-extension strength of the fractured and non-fractured leg improved significantly in both groups from baseline to 14-week follow-up. Between-group difference of the fractured leg was insignificant 0.11 (95%Cl 0.25;0.48) Nm/kg in favor of the INT (Table2). The median percentage change in knee-extension strength of fractured leg was 178% (41263) for INT and 50% (20173) for CON (*p*=0.28). Corresponding, between-group difference of the non-fractured leg was insignificant 0.16 (95% CI -0.05;0.36) Nm/Kg, with a median percentage change in knee-extension strength of non-fractured leg of 31% (1253%) for INT and 8% (033) for CON (*p*=0.04). Per protocol analysis of participants adherent to exercise (*n*=18), injections (*n*=16) and to both exercise and injections (*n*=14) are presented in Table2. Between-group differences in knee-extension strength increased in all 3 analysis for both fractured and non-fractured leg.

**Table 2:** Analysis of primary outcome, knee-extension strength (*n*=21)

Primary outcome	Baseline Mean (SD)		Follow-up Mean (SD)		Within-group difference Mean (95% CI)		Between-group difference Mean (95% CI)
**Modified intention-to-treat**							
	**INT** **(*n*=11)**	**CON** **(*n*=10)**	**INT** **(*n*=11)**	**CON** **(*n*=10)**	**INT** **(*n*=11)**	**CON** **(*n*=10)**	
Strength, Fractured (Nm/kg)	0.56 (0.38)	0.72 (0.36)	1.17 (0.46)	1.23 (0.39)	0.61 (0.34;0.88)	0.50 (0.21;0.79)	0.11 (0.25; 0.48)
Strength, non-fractured (Nm/kg)	1.07 (0.45)	1.27 (0.26)	1.35 (0.39)	1.40 (0.39)	0.28 (0.20;0.37)	0.13 (0.07;0.32)	0.16 (0.05; 0.36)
Strength, fractured % non-fractured (%)	50.5 (21.6)	59.4 (31.0)	84.6 (15.4)	89.1 (16.2)	34.1 (16.5;51.6)	29.7 (9.0;50.3)	4.4 (20.7; 29.5)
**Per protocol**							
**Exercise**^**a**^	**(*n*=8)**	**(*n*=10)**	**(*n*=8)**	**(*n*=10)**	**(*n*=8)**	**(*n*=10)**	
Strength, Fractured (Nm/kg)	0.62 (0.42)	0.73 (0.37)	1.35 (0.40)	1.23 (0.39)	0.72 (0.42;1.03)	0.50 (0.21;0.79)	0.23 (0.16; 0.61)
Strength, non-fractured (Nm/kg)	1.15 (0.50)	1.27 (0.26)	1.45 (0.40)	1.40 (0.39)	0.29 (0.19;0.40)	0.13 (0.07;0.32)	0.17 (0.04; 0.37)
**Injections**	**(*n*=7)**	**(*n*=9)**	**(*n*=7)**	**(*n*=9)**	**(*n*=7)**	**(*n*=9)**	
Strength, Fractured (Nm/kg)	0.36 (0.17)	0.69 (0.37)	1.14 (0.45)	1.25 (0.40)	0.78 (0.47;1.09)	0.56 (0.26;0.85)	0.22 (0.17;0.61)
Strength, non-fractured (Nm/kg)	1.00 (0.48)	1.29 (0.26)	1.33 (0.39)	1.39 (0.41)	0.33 (0.23;0.42)	0.10 (0.11;0.31)	0.22 (0.01;0.44)^*^
**Exercise+injections**	**(*n*=5)**	**(*n*=9)**	**(*n*=5)**	**(*n*=9)**	**(*n*=5)**	**(*n*=9)**	
Strength, Fractured (Nm/kg)	0.38 (0.17)	0.69 (0.37)	1.27 (0.48)	1.25 (0.40)	0.89 (0.49;1.29)	0.56 (0.26;0.85)	0.34 (0.10;0.77)
Strength, non-fractured (Nm/kg)	1.05 (0.57)	1.29 (0.26)	1.36 (0.47)	1.39 (0.41)	0.32 (0.18;0.46)	0.10 (0.11;0.31)	0.22 (0.07;0.50)

Cases removed if adherence below 75% for exercise (pre-defined) and 80% for injections (pre-defined as 100% but changed to 80% as this was reached by only one missing injection)

*INT* intervention (anabolic group), *CON* control group

^a^ 3 participants non adherent to exercise due to Covid-19 lock-down

^*^ *P*=0.046 (Sattertwaite due to unequal variance)

### Secondary outcomes

No significant between-group differences were identified for any of the secondary performance measures or patient reported outcomes (eTable4, additionalfile1). Increase in plasma testosterone for the INT was median 3.9 (1.2;7.5) nmol/l and for the CON 0.15 (0;0.4) nmol/l, median between-group difference was 3.7nmol/l (*p*=0.04, Wilcoxon rank sum test). Otherwise, no significant differences were identified for any of the other hormone parameters, cholesterol, CRP or body composition (eTable4, additionalfile1). Physical activity monitored after ceased intervention showed an in-significant between-group difference 0.68 (1.42; 2.79) hours/day in upright time (eTable5, additionalfile1).

### Adverse events

Fifty-seven adverse events were registered, 27 in INT and 30 in CON. Fifty-four events were categorized as non-serious and 3 as serious (1: Myocardial infarction (preexisting coronary stenosis, treated with stent), 2: 24-h hospital stay because of hip fracture-related pain, 3: Extended hospitalization due to infection). Of the 57 events 39 were categorized as non-related (eTable6, additionalfile1) and 18 as related (Adverse reactions, Table3), the summary of product characteristics for Deca-Durabolin was used as reference.

**Table 3:** Adverse reactions by group^a^

Event	INT	CON
Increased lever parameters	1	2
Increased cholesterol parameters (+triglyceride)	3	2
Increased sweating	1	1
Nausea	1	1
Edema + (foot ulcer, upper side from edema)	1	
Rasch		1
Increased PSA	1	
Hirsutism	1	
Increased blood pressure	1	
Increased libido		1
Total	10	8

^a^Categorized as potentially related to anabolic steroid prior to un-blinding

## Discussion

The HIP-SAP pilot trial is to our knowledge the first trial investigating the feasibility and preliminary effect of anabolic steroid in addition to physiotherapy and protein-rich nutritional supplement in rehabilitation following hip fracture surgery. The trial provides important knowledge on feasibility, that will help inform future trials emphasizing the difficulties to perform interventional studies in this frail patient population. Acute hospital recruitment was difficult and seem to be a major limitation in the current trial design. On the contrary, adherence to injections and exercise was high, 87 and 91% respectively, indicating excellent acceptability of the intervention. Although inconclusive due to the small sample size, promising tendencies were seen for the addition of anabolic steroid on primary outcome of knee-extension muscle strength.

### Recruitment

The inclusion rate of approximately 1.8 patients pr. month was low and less than half of what we expected based on a previous RCT [1], but comparable to trials with similar interventions [28,30]. The two most dominant reasons for non-eligibility were not home-dwelling (18%) and cognitive dysfunction (16%). Of eligible patients 41% was included. The most frequent reason to decline participation was feeling Overwhelmed and stressed by situation. It is well known that recruitment efficacy declines with increasing age of the population, and often inclusion targets are not met in populations of acute hospitalized geriatric patients [31,33]. For hip fracture patients the first postoperative days are characterized by fatigue, pain, dullness from medication. They experience a severe decline in mobility, increased dependency and many are concerned with life after the fracture [34, 35]. The highly accelerated acute hospital stay (median 8days), left few post-operative days and little time for inclusion and outcome assessment, in addition to the many standard clinical procedures during admission. Therefore, being in a stressful situation with little time to consider participation might have impeded recruitment, and some patients asked for the possibility to consider participation and decide when back home. Positively and contrary to our anticipations, worries about adverse events related to trial medication, was not a major issue for this population.

In this trial, rather conservative eligibility criteria were applied, since the use of anabolic steroid is novel in this multimorbid patient group. Less restrictive eligibility criteria could be considered in future trials, e.g. patients residing at nursing homes or those with mild cognitive dysfunctions, might be able to participate, when situated in known surroundings, and with the right support [36]. Postponing inclusion and Consent by proxy should be considered to increase inclusion rate. Acute illness as a result of surgery e.g. renal impairment may also be modified by later inclusion.

### Adherence

Adherence to injections and physiotherapy with strength training was excellent and interpreted as an expression of good acceptability of the interventions. During the physiotherapy intervention, patients were able to progressively increase loads in the strength exercises throughout the trial period, supporting acceptability of intervention. On the contrary, adherence to the nutritional supplement was lower than expected (61%), but comparable to similar trials [37, 38]. The patients described loss of appetite, nausea and disliking taste as the most frequent barriers for consumption. Loss of appetite and nausea could be a consequence of surgical stress and opioid treatment and not necessarily related to the product. Malnutrition is a modifiable potential risk factor for poor outcomes following hip fracture surgery [22, 37]. Serum albumin concentration is the most commonly used serum marker of malnutrition, in which patients are considered to be malnourished when serum albumin concentrations are <35g/L [39]. Given the albumin levels at baseline (mean of 25.9g/L (2.8)) all patients were malnourished (eTable7, additionalfile1). At follow-up albumin values had increased to 39.3g/L (3.3). None of the patients were malnourished when assessed by MNA-SF at baseline and only 5 patients were at risk of malnutrition. A consideration for future trials, is to individualize type of supplement and provided it only for patients at nutritional risk.

The retention to hospital controls was excellent, although some patients experienced getting out of the house and the transportation as strenuous. Home visits could be considered in future trials, or maybe a different form of anabolic steroid preparation, of which the patients or home care could administer.

### Preliminary effect of primary and secondary outcomes

Although inconclusive, some tendencies were seen for the addition of anabolic steroid on the primary outcome of knee-extension muscle strength and in favor of INT in the modified intention-to-treat analysis. Also, in per protocol analyses, between-group differences for participants adherent to the anabolic steroid almost reached significance for the non-fractured leg (*p*=0.046) in favor of INT.

Overall, a tendency of larger strength improvements was seen in the non-fractured leg compared to the fractured leg, probably due to less variance caused by trauma and surgery related pain and edema [2, 40]. However, pain was not a limiting factor during strength-testing. At baseline only 2 in INT and 4 in CON expressed moderate to severe pain (VRS>1), at follow-up no patients reported moderate to severe pain (eTable8, additionalfile1).

Disappointingly, no significant between-group difference was identified for secondary performance- or patient reported measures, while significant positive within-group changes were seen for gait, mobility and fear of falling, as expected. No within-group change were seen for hand grip strength, QoL, fatigue, depression, as in line with similar previous studies [23, 41, 42]. Testosterone levels for both genders were very low at baseline, but as expected higher levels of testosterone were found in the INT at follow-up.

Several studies report decline in BMD following hip fracture [43,45]. Positively, INT showed a significant increase in whole body BMD of 0.019g/cm^2^ whereas it decreased for CON (0.015g/cm^2^). A previous study also showed positive effect of 6months treatment with nandrolone on LBM in elderly female hip fracture patients [46], but no significant between-group differences were seen for measures of LBM in the present study. The lack of effect is probably explained by short treatment period as well as low sample size. Weight loss in both groups can to some extent be explained by post-surgery edema and fluid retention at baseline, which is in line with the reduction in total LBM and in accordance with previous studies showing a reduction in LBM of 3.46.4% from 3days after surgery until 2months [43, 44].

### Adverse events

Adverse events and adverse reactions were equally distributed in the two groups. Female hirsutism following anabolic treatment have been reported in a previous study [30] and was a concern. Only one woman reported a slight increase in facial hair growth on the chin, but she used to shave prior to trial, and was not concerned with it, nonetheless injections were stopped as she was bothered with increased perspiration. Three patients had increased liver parameters, 2 (one in each group) with levels above the safety threshold, and medication was ceased. In both cases parameters were normalized within 2weeks.

### Strength and limits

A methodological strength is the trial design being a randomized blinded placebo-controlled trial. Further the deliverance of the exercise intervention mimics everyday practice, with initiation of the intervention during admission in the acute setting and continuing in the municipality for approximately 12weeks. The content of the physiotherapy intervention is similar to the existing standard rehabilitation offered by the municipalities, which increases external validity and could ease implementation. Further, we consider it at a major strength, that progressive strength training were demonstrated to be feasible and with high adherence, since strength training is crucial for patients with hip fracture as loss of muscle strength is a serious consequence of the fracture, but also since preexisting sarcopenia is prevalent in this population [47].

The trial is limited by the low inclusion rate, as we were not able to reach the calculated number needed in the trial. Thus, we were not able to conclude for or against the intervention. Further, the participants included were younger and had a higher prefracture functional level compared to the general population of older hip fracture patients admitted from own home [48]. Consequently, generalizability of the results will be limited to a similar population. However, even the fitter hip fracture patients have strength deficits and potential for improving strength and function. In a recently published study, we found that almost half of hip fracture patients were classified as probable sarcopenic using cut-points for knee extension strength of the non-fractured leg and hand grip strength [49]. Furthermore, a study by Dyer et al. [7] showed that among the fitter patients, only 4044% recovered their pre-fracture mobility independence.

Another limitation is not involving participants in the design of the study, which was not possible due to limited resources and the complexity of the trial.

## Conclusion

Early inclusion after hip fracture surgery to this cross-continuum drug trial investigating the effect of anabolic steroid during rehabilitation seemed non-feasible, primarily due to a low inclusion rate. The trial illustrates the complexity of challenges related to carrying out randomized controlled trials in patients with hip fracture. Although inconclusive due to the small sample size, promising tendencies were seen for the addition of anabolic steroid.

## Supplementary Information

**Additional file 1.** Supplementary material (eMethod and eTables18).

## Data Availability

MTK owns data, all authors have full access to the dataset. The datasets analyzed during the current study are available from the corresponding author on reasonable request.

## Abbreviations

INT: Intervention group
CON: Control group
RM: Repetition maximum
ICH-GCP: International conference on harmonisation - good clinical practice
VRS: Verbal rating scale
NMS: New mobility score
NYHA: New York Heart Association
PSA: Prostate specific antigen
HGS: Hand-grip strength
DEXA: Dual x-ray absorptiometry
BDM: Bone mineral density
LBM: Lean body mass
MNA-SF: Mini nutritional assessment short form
TUG: Timed up and go
DEMMI: The de mortons mobility index
HRQoL: Health related quality of life
Short FES-I: Short falls efficacy scale international
GDS-15: Geriatric depression scale
CRF: Case report form
REDCap: Research electronic data capture
GCP: Good clinical practice
SOP: Standard operating procedure
AE: Adverse event
AR: Adverse reaction
