# Supplementary material for: Feasibility and preliminary effect of anabolic steroids in addition to strength training and nutritional supplement in rehabilitation of patients with hip fracture: a randomized controlled pilot trial (HIP-SAP1 trial)
Source: BMC Geriatr. 2021 May 20;21:323. doi: 10.1186/s12877-021-02273-z (PMC8136760; doi:10.1186/s12877-021-02273-z)
Supplement: Supplementary file 1 — Additional file 1. Supplementary material (eMethod and eTables18). [file 12877_2021_2273_MOESM1_ESM.docx]

**Supplementary material**

**Feasibility and preliminary effect of strength training, nutritional supplement and anabolic steroids in rehabilitation of patients with hip fracture: A randomized controlled pilot trial (HIP-SAP1 trial)**

Signe Hulsbæk PT, MPH^1^; Thomas Bandholm MSc, PhD^1,2,3,6^; Ilija Ban MD, PhD^2,6^; Nicolai Bang Foss MD, PhD ^4,6^; Jens-Erik Beck Jensen MD, PhD ^5,6^; Henrik Kehlet MD, PhD ^7^; Morten Tange Kristensen PT, PhD ^1,2,6^.

1. *Physical Medicine and Rehabilitation Research – Copenhagen (PMR-C), Department of Physiotherapy, Copenhagen University Hospital, Hvidovre, Denmark*
2. *Department of Orthopedic Surgery, Copenhagen University Hospital, Hvidovre, Denmark*
3. *Department of Clinical Research, Copenhagen University Hospital, Hvidovre, Denmark*
4. *Department of Anesthesiology, Copenhagen University Hospital, Hvidovre, Denmark*
5. *Department of Endocrinology, Copenhagen University Hospital, Hvidovre, Denmark*
6. *Department of Clinical Medicine, University of Copenhagen, Denmark*
7. *Section for Surgical Pathophysiology, Copenhagen University Hospital, Rigshospitalet, Denmark*

**Content:**

**eMethods: Description of secondary outcome measures with references**

**eTable 1: Numbers and reasons for non-participation**

**eTable 2: Patient reported reasons for declining to participate**

**eTable 3: Training load progression in percentage**

**eTable 4: Analysis of secondary outcomes**

**eTable 5: Physical activity (Active Pal, measured at week 12, after ceased intervention)**

**eTable 6: Events categorized as non-related**

**eTable 7: Other blood parameters discussed in main text**

**eTable 8: Pain during muscle strength testing and TUG**

**eMethods:**

**Description of secondary outcome measures**

Unless stated otherwise, the change in values are measured from baseline until 14 weeks.

**Performance measures**

Maximal isometric knee-extension strength (Nm/Kg) in the non-fractured limb. Maximal isometric knee-extension strength (Nm/Kg) in the fractured limb in % of the non-fractured limb. Hand-grip strength (HGS) measured in Kg. in the dominant hand, using a digital handheld dynamometer (Saehan Grip, DHD-1). Gait speed in m/s was assessed using the 10-meter fast speed walking test, standing start (10mWT) ^1^. Timed up and go test (TUG) measured in seconds (and 10mWT) was performed preferable using a 4-wheeled rollator if not possible the walking aid the patient was able to manage independently ^2,3^. The de Mortons Mobility Index (DEMMI), score range from 0 to 100, 100 representing the highest level of mobility ^4–6^. Physical activity as sedentary time (lying/sitting) and upright time (standing/walking), steps and transfers is measured using a body-worn accelerometer activity monitor (ActivePAL) ^7^. The patient wore the monitor for one week following the 12-week control.

**Patient reported outcomes**

Nutrition screening using the Mini Nutritional Assessment Short Form (MNA-SF), range 0-14, high scores indicating better nutritional status ^8,9^. Functional level was assessed by the modified New Mobility Score (NMS), range 0-9, higher score indicating higher independence (baseline assessment refers to prefracture status) ^10–12^. EQ-5D-3L assessing Health Related Quality of Life (HRQoL), baseline assessment refers to the time prior to the fracture ^13–15^. Hip fracture related pain at rest and during outcome assessment is evaluated by Verbal Rating Scale (VRS), 0-4 ^16^. The Short Falls Efficacy Scale-International (Short FES-I) measured fear of falling (score range from 7–28, higher scores indicating a higher fear of falling) ^17,18^. Fatigue was assessed using the SF36 vitality subscale, consisting of 4 items, score range from 0-100, high score defines a more favorable health state, baseline assessment refers to pre-fracture state ^19,20^. Depression is assessed using the Geriatric Depression Scale (GDS-15), administered as an interview, score range 0-15, baseline assessment refers to pre-fracture status ^21,22^.

**Dual x-ray absorptiometry (DEXA)**

Bone mineral density (BMD) of total body, total hip, femoral neck and lumbar spine. BMD is expressed in g/cm^2^ and T-score was registered. Lean body mass (LBM) of total body, legs bilaterally and arms bilaterally expressed in kg. Total body fat mass expressed in kg.

**Blood tests**

Total testosterone (nmol/l), Luteinizing hormone, LH (IU/l), Follicle-stimulating hormone, FSH (IU/l), Sex hormone binding globulin, SHBG (nmol/l). Lipid profile (Total cholesterol, HDL cholesterol, LDL cholesterol, triglyceride), mmol/l. C-reactive protein (CRP), mg/l.

**References**

1. Overgaard J. Feasibility of progressive strength training shortly after hip fracture surgery. *World J Orthop*. 2013;4(4):248. doi:10.5312/wjo.v4.i4.248

2. Kristensen MT, Henriksen S, Stie SB, Bandholm T. Relative and absolute intertester reliability of the timed up and go test to quantify functional mobility in patients with hip fracture. *J Am Geriatr Soc*. 2011;59(3):565-567. doi:10.1111/j.1532-5415.2010.03293.x

3. Bloch ML, Jønsson LR, Kristensen MT. Introducing a Third Timed Up &amp; Go Test Trial Improves Performances of Hospitalized and Community-Dwelling Older Individuals. *J Geriatr Phys Ther*. 2017;40(3):121-126. doi:10.1519/JPT.0000000000000080

4. de Morton NA, Harding KE, Taylor NF, Harrison G. Validity of the de Morton Mobility Index (DEMMI) for measuring the mobility of patients with hip fracture during rehabilitation. *Disabil Rehabil*. 2012;35(June 2012):1-9. doi:10.3109/09638288.2012.705220

5. de Morton NA, Davidson M, Keating JL. Validity, responsiveness and the minimal clinically important difference for the de Morton Mobility Index (DEMMI) in an older acute medical population. *BMC Geriatr*. 2010;10:72. doi:10.1186/1471-2318-10-72

6. Hulsbæk S, Larsen RF, Rosthøj S, Kristensen MT. The Barthel Index and the Cumulated Ambulation Score are superior to the de Morton Mobility Index for the early assessment of outcome in patients with a hip fracture admitted to an acute geriatric ward. *Disabil Rehabil*. Published online January 15, 2018:1-9. doi:10.1080/09638288.2018.1424951

7. Taraldsen K, Askim T, Sletvold O, et al. Evaluation of a Body-Worn Sensor System to Measure Physical Activity in Older People With Impaired Function. *Phys Ther*. 2011;91(2):277-285. doi:10.2522/ptj.20100159

8. Koren-Hakim T, Weiss A, Hershkovitz A, et al. Comparing the adequacy of the MNA-SF, NRS-2002 and MUST nutritional tools in assessing malnutrition in hip fracture operated elderly patients. *Clin Nutr*. 2016;35(5):1053-1058. doi:10.1016/j.clnu.2015.07.014

9. Helminen H, Luukkaala T, Saarnio J, Nuotio MS. Predictive value of the mini-nutritional assessment short form (MNA-SF) and nutritional risk screening (NRS2002) in hip fracture. *Eur J Clin Nutr*. 2019;73(1):112-120. doi:10.1038/s41430-018-0267-y

10. Kristensen MT, Kehlet H. Most patients regain prefracture basic mobility after hip fracture surgery in a fast-track programme. *Dan Med J*. 2012;59(6):A4447.

11. Parker MJ, Palmer CR. A new mobility score for predicting mortality after hip fracture. *J Bone Joint Surg Br*. 1993;75(5):797-798.

12. Kristensen M, Bandholm T, Foss N, Ekdahl C, Kehlet H. High inter-tester reliability of the new mobility score in patients with hip fracture. *J Rehabil Med*. 2008;40(7):589-591. doi:10.2340/16501977-0217

13. Tidermark J, Bergström G. Responsiveness of the EuroQol (EQ-5D) and the Nottingham Health Profile (NHP) in elderly patients with femoral neck fractures. *Qual Life Res*. 2007;16(2):321-330. doi:10.1007/s11136-006-9004-4

14. Parsons N, Griffin XL, Achten J, Costa ML. Outcome assessment after hip fracture. *Bone Joint Res*. 2014;3(3):69-75. doi:10.1302/2046-3758.33.2000250

15. Haywood KL, Brett J, Tutton E, Staniszewska S. Patient-reported outcome measures in older people with hip fracture: a systematic review of quality and acceptability. *Qual Life Res*. 2017;26(4):799-812. doi:10.1007/s11136-016-1424-1

16. Bech RD, Lauritsen J, Ovesen O, Overgaard S. The Verbal Rating Scale Is Reliable for Assessment of Postoperative Pain in Hip Fracture Patients. *Pain Res Treat*. 2015;2015:1-7. doi:10.1155/2015/676212

17. Kempen GIJM, Yardley L, Van Haastregt JCM, et al. The Short FES-I: a shortened version of the falls efficacy scale-international to assess fear of falling. *Age Ageing*. 2008;37:45-50. doi:10.1093/ageing/afm157

18. Visschedijk JHM, Terwee CB, Caljouw MAA, Spruit-van Eijk M, van Balen R, Achterberg WP. Reliability and validity of the Falls Efficacy Scale-International after hip fracture in patients aged ≥ 65 years. *Disabil Rehabil*. 2015;37(23):2225-2232. doi:10.3109/09638288.2014.1002573

19. Ware JE, Sherbourne CD. The MOS 36-item short-form health survey (SF-36). I. Conceptual framework and item selection. *Med Care*. 1992;30(6):473-483.

20. Neuberger GB. Measures of fatigue: The Fatigue Questionnaire, Fatigue Severity Scale, Multidimensional Assessment of Fatigue Scale, and Short Form-36 Vitality (Energy/Fatigue) Subscale of the Short Form Health Survey. *Arthritis Rheum*. 2003;49(S5):S175-S183. doi:10.1002/art.11405

21. Djernes, J.K.; Kvist, E.; Olesen, F.; Munk-Jørgensen, Povl; Gulmann NC. Validering af dansk oversættelse af Geriatric Depression Scale-15 som screeningsredskab for depression blandt hjemmeboende svage ældre. *Ugeskr Laeger*. 2004;166(10):905-909.

22. Yesavage JA, Brink TL, Rose TL, et al. Development and validation of a geriatric depression screening scale: a preliminary report. *J Psychiatr Res*. 17(1):37-49.

| **eTable 1: Numbers and reasons for non-participation (n=688)** | | |
| --- | --- | --- |
| **Reason** | | **Numbers (%)** |
| **1** | Age <60 years | 64 (8.9) |
| **2** | Not Speaking and understanding Danish or having a Danish Social Security Number. | 21 (2.9) |
| **3** | Unable to give informed consent (see point 11) | - |
| **4** | Residing at nursing home/24-hour rehabilitation prefracture. | 130 (18.1) |
| **5** | No Independent pre-fracture indoor walking ability (NMS<2) | 6 (0.8) |
| **6** | Weight-bearing restrictions | 22 (3.1) |
| **7** | Multiple fractures | 26 (3.6) |
| **8** | Active cancer or suspected pathological fracture | 33 (4.6) |
| **9** | Patients unable/unwilling to cooperate for testing and rehabilitation | 38 (5.3) |
| **10** | Planned/elective hospitalization within the trial period. | 4 (0.6) |
| **11** | Cognitive dysfunction (disoriented, dementia, active delirium) | 117 (16.3) |
| **12** | Uncontrolled blood pressure (systolic > 150 mmHg, or diastolic > 100 mmHg) | 5 (0.7) |
| **13** | Heart disease (peri-, myo- or endocarditis) | 0 |
| **14** | History of stroke with motor disability. | 22 (3.1) |
| **15** | Heart failure (NYHA class III and IV) | 3 (0.4) |
| **16** | Kidney failure or renal impairment | 40 (5.6) |
| **17** | Abnormal liver function tests or history of hepatic tumor | 3 (0.4) |
| **18** | Elevated hematocrit ≥ 50% | 0 |
| **19** | History of breast or prostate cancer | 25 (3.5) |
| **20** | Abnormally elevated serum PSA assessed at the 3-week control | 1 (0.1) |
| **21** | Allergic to ingredients in the Deca-Durabolin solution or the nutritional supplement. | 1 (0.1) |
| **22** | Transferred to another department | 3 (0.4) |
| **23** | Living outside uptake area | 66 (9.2) |
| **24** | Not drinking p-drink (prior to loosing this criteria) | 1 (0.1) |
| **25** | Declining participation | 41 (5.7) |
| **26** | Estrogen tablet treatment | 1 (0.1) |
| **27** | Acute postoperative illness (nonspecific to other criteria’s) | 9 (1.3) |
| **28** | Admitted after surgery in another hospital | 2 (0.3) |
| **29** | Fast discharge | 3 (0.4) |
| **30** | Died in hospital | 1 (0.1) |
| Note. NMS: New Mobility Score, NYHA: New York Heart Association; PSA: Prostate-specific antigen | | |

| **eTable 2: Patient-reported reasons for declining participation although fulfilling inclusion criteria (n=41)** | | |
| --- | --- | --- |
| **Reasons** | **Primary** | **Secondary** |
| Overwhelmed and stressed by situation | 15 | 1 |
| Extra hospital controls | 6 | 3 |
| Disliking principle of randomization | 1 | 4 |
| Personal factors (e.g. a sick relative, job) | 6 | - |
| Worries related to adverse events | 2 | 2 |
| Disliking protein | - | 2 |
| Generally abstaining from medication | 3 | 1 |
| Relatives did not want patient to participate | 3 | - |
| No eyeglasses – couldn’t read information and consent. | 1 | - |
| Did not want rehabilitation | - | 1 |
| Did not want information | 4 | - |
| **Total** | **41** | **14** |

| **eTable 3: Progression in training loads in percentage ^a^** | | | |
| --- | --- | --- | --- |
|  | **n** | **Median (q1; q3)**  **% Increase** | **Range**  **% Increase** |
| **Session 2 – 4,**  **15 repetition maximum** |  |  |  |
| Knee extension, fractured | 18 | 31.5 (10; 67) | 0; 250 |
| Knee extension, non-fractured | 9 ^b^ | 14 (0; 18) | 0; 63 |
| Leg press, bilateral | 18 | 20 (9; 58) | -17; 133 |
| **Session 5 – 8,**  **12 repetition maximum** |  |  |  |
| Knee extension, fractured | 18 | 18 (0; 59) | -41; 80 |
| Knee extension, non-fractured | 9 | 17 (9; 33) | 6; 65 |
| Leg press, bilateral | 18 | 7.5 (0; 17) | 0; 100 |
| **Session 9 –**  **10 repetition maximum** |  |  |  |
| Knee extension, fractured | 18 | 50 (7; 73) | -20; 140 |
| Knee extension, non-fractured | 9 | 23 (14; 38) | -8; 67 |
| Leg press, bilateral | 18 | 38.5 (30; 44) | -38; 78 |
| ^a^ Different weight training machines were used at the 9 outpatient settings. Accordingly, the percentage progression of loads within the different repetition maximum sessions are reported.  ^b^ In the first version of the exercise log, it was only possible to register load in knee extension on the fractured leg although both legs were trained during the entire study. This was changed after the first participant, but not all rehabilitation centers succeeded in registering on both legs. | | | |

| **eTable 4:** **Analysis of secondary outcomes (n=21)** | | | | | | | |
| --- | --- | --- | --- | --- | --- | --- | --- |
|  | **Baseline**  **Mean (SD)** | | **Follow-up**  **Mean (SD)** | | **Within-group difference**  **Mean (95% CI)** | | **Between-group difference Mean (95% CI)** |
| **Performance** | **INT**  **n=11** | **CON**  **n=10** | **INT**  **n=11** | **CON**  **n=10** | **INT**  **n=11** | **CON**  **n=10** |  |
| Handgrip strength (Kg) | 27.6 (10.6) | 26.8 (5.8) | 28.4  (9.7) | 26.4  (6.2) | 0.9  (-0.9; 2.6) | -0.4  (-2.7; 1.9) | 1.3  (-1.4; 4.0) |
| TUG (s) | 27.7  (8.5) ^a^ | 31.4 (14.6) | 8.4  (2.2) | 8.8  (2.5) | 19.5  (14.2; 24.8) ^a^ | 22.6  (12.3; 33.0) | -3.13  (-13.9; 7.7) |
| 10mwt (m/s) | 0.63 (0.28) | 0.59 (0.25) ^b^ | 1.35  (0.31) | 1.33 (0.32) | 0.73  (0.58; 0.87) | 0.72  (0.38; 1.06) | 0.004  (-0.35; 0.35) |
| DEMMI  (0-100) | 45.2  (6.8) | 44.8  (8.2) | 77.4  (11.5) | 78.1 (11.4) | 32.2  (24.8; 39.6) | 33.3  (25.9; 40.7) | -1.1  (-10.9; 8.7) |
| **Patient reported** | | | | | | | |
| NMS (0-9) | 8.6  (0.8) | 8.5  (1.1) | 7.9  (1.3) | 8.3  (1.2) | -0.7  (-1.6; 0.1) | -0.2  (-0.7; 0.3) | -0.5  (-1.5; 0.4) |
| EQ-5D VAS  (0-100) | 82.7 (17.7) | 84.3 (17.9) | 82.6 (17.7) | 81.5 (12.3) | -0.1  (-10.2; 10.0) | -2.8  (-14.8; 9.2) | 2.71  (-11.8; 17.2) |
| EQ-5D-3L  Index Score | 0.88 (0.15) | 0.90 (0.14) | 0.85 (0.14) | 0.78 (0.14) | -0.03  (-0.14; 0.08) | -0.12  (-0.22; -0.01) | 0.08  (-0.06; 0.23) |
| Short FES-I  (7-24) | 12.6 (4.8) | 13.9 (4.0) | 8.8  (2.5) | 8.9  (2.6) | -3.8  (-6.6; -1.0) | -5.0  (-7.0; -3.0) | -1.2  (4.4; -2.1) |
| Fatigue (0-100) | 75.5 (19.7) | 67.5 (16.4) | 75.0 (24.2) | 70.5 (24.1) | -0.5  (-8.5; 7.6) | 3.0  (-10.3; 16.3) | -3.45  (-17.6; 10.7) |
| GDS (0-15) | 1.4  (3.3) ^b^ | 1.2  (1.0) ^b^ | 1.9  (4.0) ^a^ | 2.2  (3.1) | 0.3  (-0.6; 1.3) ^b^ | 0.1  (-0.5; 0.7) ^b^ | 0.2  (-0.8; 1.2) |
| MNA-SF (0-14) | 13  (1.6) | 12.6 (2.3) | 12.7  (1.4) | 11.7 (2.4) | -0.3  (-1.3; 0.7) | -0.9  (-2.9; 1.1) | 0.6  (-1.4; 2.7) |
| **Blood test** | | | | | | | |
| Testosterone, nmol/l | 0.66 (0.7) | 1.38 (3.1) | 5.82  (6.5) | 2.75 (5.1) | 5.15  (0.90; 9.41) | 1.37  (-0.97; 3.71) | 3.4  (-0.90; 8.47) ^c^ |
| LH, IU/I | 11.7 (17.7) | 9.2  (8.3) | 25.7 (17.3) | 28.3 (14.6) | 14.0  (0.4; 27.5) | 19.1  (9.6; 28.6) | -5.14  (-20.9; 10.6) |
| FSH, IU/I | 24.0 (21.8) | 25.4 (13.9) | 54.8 (41.0) | 56.8 (30.2) | 30.8  (14.6; 47.0) | 31.3  (16.0; 46.6) | -0.53  (-21.4; 20.4) |
| SHBG, nmol/l | 50.6 (18.6) | 72.8 (36.4) | 67.0 (36.8) | 97.2 (54.7) | 16.4  (-4.0; 36.7) | 24.3  (-4.7; 53.4) | -7.97  (-40.5; 24.5) |
| Cholesterol total, mmol/l | 3.3  (0.6) | 3.4  (0.7) | 5.2  (1.0) | 4.8  (1.0) | 1.9  (1.2; 2.6) | 1.4  (0.8; 2.1) | 0.47  (-0.39; 1.33) |
| HDL, mmol/l | 1.2  (0.3) | 1.2  (0.5) | 1.6  (0.5) | 1.8  (0.7) | 0.4  (0.2; 0.8) | 0.6  (0.4; 0.8) | -0.18  (-0.49; 0.12) |
| LDL, mmol/l | 1.4  (0.5) | 1.4  (0.7) | 2.7  (1.2) ^a^ | 2.3  (0.9) | 1.2  (0.5; 2.0) | 0.9  (0.4; 1.4) | 0.37  (-0.44; 1.18) |
| Triglycerid, mmol/l | 1.52 (0.27) | 1.72 (0.48) | 2.08 (1.24) | 1.60 (0.75) | 0.56  (-0.17; 1.30) | -0.12  (-0.49; 0.24) | 0.69  (-0.10; 1.47) ^d^ |
| CRP, mg/l | 143.1 (102.9) | 91.5 (47.1) | 7.7  (11.3) | 2.8  (3.9) | -135.4  (-202.0; -68.7) | -88.7  (122.8; -54.6) | -46.7  (-118.2; 24.9) |
| **Body composition** | | | | | | | |
| Weight, kg | 75.9 (12.1) | 74.6 (16.7) | 71.9 (11.8) | 72.3 (16.9) | -3.9  (-6.2; -1.6) | -2.3  (-4.2; -0.3) | -1.66  (-4.50; 1.19) |
| BMI | 27.5 (4.4) | 25.8 (5.0) | 26.0  (3.8) | 25.2 (5.0) | -1.4  (-2.4; -0.5) | -0.6  (-1.4; 0.2) | -0.83  (-1.97; 0.31) |
| BMD total, g/cm2 | 1.20 (0.10) | 1.16 (0.16) | 1.22  (0.11) | 1.15 (0.13) | 0.019  (0.001; 0.037) | -0.015  (-0.055; 0.025) | 0.034  (-0.008; 0.076) |
| LBM total, kg | 47.2 (9.4) | 47.4 (10.6) | 45.2  (8.9) | 44.1 (10.3) | -2.0  (-3.5; -0.5) | -3.3  (-4.3; -2.3) | 1.3  (-0.44; 2.97) |
| LBM right arm, kg | 2.2  (0.6) | 2.3  (0.8) | 2.3  (0.6) | 2.3  (0.9) | 0.1  (-0.1; 0.2) | 0  (-0.1; 0.1) | 0.1  (-0.10; 0.29) |
| LBM left arm, kg | 2.0  (0.6) | 2.1  (0.8) | 2.2  (0.7) | 2.1 (0.7) | 0.2  (0.1; 0.4) | 0.01  (-0.2; 0.2) | 0.2  (-0.01; 0.42) |
| LBM fract. leg, kg | 8.5  (2.1) | 8.4  (2.0) | 7.0  (1.5) | 6.7 (1.7) | -1.5  (-2.2; -0.8) | -1.7  (-2.1; -1.2) | 0.12  (-0.67; 0.91) |
| LBM non-fract.leg, kg | 7.0  (1.8) | 7.0  (1.6) | 7.0  (1.5) | 6.8 (1.7) | 0.01  (-0.3; 0.3) | -0.2  (-0.5; 0.1) | 0.17  (-0.25; 0.59) |
| LBM summed upper extr. | 4.2  (1.2) | 4.4  (1.6) | 4.5  (1.3) | 4.4 (1.5) | 0.31  (0.05; 0.57) | 0.01  (-0.30; 0.32) | 0.30  (-0.07;0.67) |
| Fat mass | 26.5 (8.9) | 27.0 (8.5) | 25.2  (7.8) | 27.1 (8.6) | -1.3  (-2.6; 0.04) | 0.1  (-1.1; 1.3) | -1.37  (-3.03; 0.29) |
| Note. TUG: Timed Up and Go test, 10mwt: 10 meter walk test, DEMMI: de Mortons Mobility Index, NMS: New Mobility Score; EQ-5D: EuroQol- 5 Domain, VRS: Verbal rating scale, Short FES-I: Short Falls Efficacy Scale-International; GDS: Geriatric Depression Scale, MNA-SF: Mini Nutritional Assessment Short Form, LH: Luteinizing hormone, FSH: Follicle-stimulating hormone, SHBG: Sex hormone binding globulin, HDL: High density lipoprotein cholesterol, LDL: Low density lipoprotein cholesterol, CRP: C-reactive protein. BMI: Body Mass Index, BMD: Bone Mineral Density, LBM: Lean body Mass.  Note. For between group differences T-test or Wilcoxon ranked sum test have been performed according to our best evaluation of normal distribution.  ^a^ n=10  ^b^ n=9  ^c^ Data is not normally distributed and a non-parametric test (Wilcoxon rank sum, exact) was performed, P=0.04  ^d^ Satterthwaite due to unequal variance | | | | | | | |

| **eTable 5: Physical activity (Active Pal, measured at week 12, after ceased intervention)** | | | |
| --- | --- | --- | --- |
|  | Intervention (n=10) ^a^  Mean (SD) | Control (n=10)  Mean (SD) | Difference  Mean (95% CL) |
| Sedentary time, hours/day | 18.3 (2.9) | 19.0 (0.9) | -0.68 (-2.78; 1.42) ^b^ ^c^ |
| Upright time, hours/day | 5.7 (2.9) | 5.0 (0.9) | 0.68 (-1.42; 2.79) ^b^ |
| Steps/day | 5952 (4673) | 6071 (2777) | -119 (-3731; 3492) ^d^ |
| Transfers/ day | 45.9 (15.4) | 45.1 (8.6) | 0.78 (-10.9; 12.5) |
| Note. Measured over 7 days (4 patients only 6 days, due to battery issues, and one patient only 2 days due to allergic reaction caused by the patch fixating the monitor).  ^a^ 1 missing due to Covid-19  ^b^ Satterthwaite due to unequal variance  ^c^ Minus indicate that intervention is less sedentary  ^d^ Precaution with interpretation due to known problem with Active Pal not recognizing “slow walking” as steps. | | | |

| **eTable 6: Un-related events** |  |  |
| --- | --- | --- |
| **Event** | **int** | **con** |
| Allergy (Quincke’s edema) | - | 1 |
| Falls | - | 3 |
| Treated for Infection cicatrice (different degrees) | 2 | 1 |
| Greenish urine | - | 1 |
| Cold (common viral infection) | 2 | 3 |
| Constipation | 1 | - |
| Edema primarily operated leg (treated with pressure socks) | 2 | - |
| Herpes zoster | 1 | - |
| Hip or knee (n=1) related pain | 1 | 5 |
| Myocardial infarction (known coronary stenosis since 2016, medically treated) | 1 | - |
| Fainting (hypotension) (same patient) | 2 | - |
| Nausea / loss of appetite | - | 2 |
| Urinary tract infection | 1 | 1 |
| Recurrence of chronic leg ulcer | - | 1 |
| Click sounds from osteosynthesis material | - | 1 |
| Feeling depressed (history of depression) | - | 1 |
| Dizziness | 1 | - |
| Stomach ulcer (history of ulcer), medically treated | 1 | - |
| Weight loss (loss of appetite, altered sense of taste had lengthy antibiotic treatment UVI) | - | 1 |
| Delayed fracture heeling | 1 | - |
| Pressure ulcer heel | 1 | - |
| Renal function slightly impaired (preexisting renal impairment) | - | 1 |
| **Total 39** | **17** | **22** |

| **eTable 7: Other blood parameters discussed in text (n=21)** | | | | | | | |
| --- | --- | --- | --- | --- | --- | --- | --- |
| **Outcome** | **Baseline** | | **Follow-up** | | **Within group difference**  **95% CI** | | **Between group diff.**  **95% CI** |
| ***Blood test*** | ***INT*** | ***CON*** | ***INT*** | ***CON*** | ***INT*** | ***CON*** |  |
| Hemoglobin, mmol/l | 6.7 (0.7) | 6.6 (0.8) | 8.8 (1.0) | 8.7 (0.3) | 2.1  (1.3;2.9) | 2.1  (1.5;2.6) | 0.02  (-0.9; 0.9) |
| Hematocrit, % | 0.32 (0.03) | 0.33 (0.04) | 0.42 (0.05) | 0.42 (0.02) | 0.10 (0.06;0.13) | 0.09 (0.06;0.11) | 0.01  (-0.03; 0.05) |
| Albumin g/L | 26.3 (2.8) | 25.4 (2.9) | 39.2 (3.9) | 39.3 (2.5) | 12.9 (10.0;15.8) | 13.9 (12.0;15.7) | -0.99  (-4.24; 2.27 |
| Note. INT: Intervention group, CON: Control group | | | | | | | |

| **eTable 8: Hip fracture-related pain during muscle strength testing and TUG.** | | | | | | | | | | | | | | | | | | | | |
| --- | --- | --- | --- | --- | --- | --- | --- | --- | --- | --- | --- | --- | --- | --- | --- | --- | --- | --- | --- | --- |
|  | **Baseline** | | | | | | | | | | **Follow-up** | | | | | | | | | |
|  | **Intervention,**  **VRS 0-4** | | | | | **Control,**  **VRS 0-4** | | | | | **Intervention,**  **VRS 0-4** | | | | | **Control,**  **VRS 0-4** | | | | |
|  | **0** | **1** | **2** | **3** | **4** | **0** | **1** | **2** | **3** | **4** | **0** | **1** | **2** | **3** | **4** | **0** | **1** | **2** | **3** | **4** |
| Pain (during fractured leg testing), numbers | 5 | 4 | 1 | 1 | 0 | 4 | 2 | 2 | 2 | 0 | 11 | 0 | 0 | 0 | 0 | 7 | 3 | 0 | 0 | 0 |
| Pain (during TUG), numbers | 3 | 4 | 3 | 0 | 0 | 3 | 6 | 1 | 0 | 0 | 11 | 0 | 0 | 0 | 0 | 6 | 4 | 0 | 0 | 0 |
| Note. VRS, Verbal Ranking Scale; 0=“no pain,” 1=“slight pain,” 2=“moderate pain,” 3=“severe pain,” 4=“unbearable pain.” | | | | | | | | | | | | | | | | | | | | |
